# Allergic Reaction to Titanium Alloy Osteosynthesis Implants: A Case Report of Three Patients

**DOI:** 10.3390/life16030388

**Published:** 2026-02-28

**Authors:** Hsuan Chou, Yen-Yu Lin, Yu-Ching Huang, Ping-Chun Yeh, Shu-Hao Chang

**Affiliations:** 1 School of Medicine, College of Medicine, Fu Jen Catholic University, New Taipei City 24205, Taiwan; eric158273@gmail.com (H.C.);; 2 Department of Pathology, Fu Jen Catholic University Hospital, Fu Jen Catholic University, New Taipei City 24352, Taiwan; 3 Department of Medical Education, Taipei Veterans General Hospital, Taipei 112201, Taiwan; ychuang48@vghtpe.gov.tw; 4 Department of Orthopedics, Fu Jen Catholic University Hospital, Fu Jen Catholic University, New Taipei City 24352, Taiwan

**Keywords:** titanium alloy, allergy, hypersensitivity, open reduction and internal fixation

## Abstract

Background: Titanium is commonly used for fracture fixation and is considered inert, but hypersensitivity reactions to titanium alloy still occur and are difficult to diagnose due to a lack of a universally accepted standard in post-orthopedic surgical patients, and other common diagnoses need to be differentiated. Case Presentation: This case report describes three patients with manifestations of allergic reactions to titanium-alloy implants after Open Reduction and Internal Fixation (ORIF) with Ti-6Al-4V plates. Three patients mainly presented with persistent localized dermatitis. During follow-up, radiographs confirmed bone unions, and there were no signs of purulent drainage, abscess formation, or systemic infection. Taken together, these findings reduce the possibility of fracture-related infection (FRI) and other etiologies. Conservative treatment was ineffective, and the patch test and histopathology in two patients respectively supported hypersensitivity reactions. After the implants were surgically removed, there were significant improvements in symptoms. Conclusion: Three cases of suspected titanium-alloy hypersensitivity were diagnosed by exclusion based on clinical history, laboratory results, and additional testing. The findings showed the importance of clinical history and the identification of signs and symptoms of allergic reactions, emphasizing the need for a standardized diagnostic process.

## 1. Introduction

Surgical implants provide stability, flexibility, and durability for patients undergoing orthopedic surgeries. However, some surgical implants include allergens that are immunogenic and might contribute to adverse tissue reactions and even implant failure [1]. Surgical implants have been reported to be associated with hypersensitivity reactions, including localized rash, itching, tenderness, edema, and stiffness, and rare systemic signs of hypersensitivity reactions, such as fever, chills, malaise, nausea, vomiting, and even anaphylactic shock, have also been reported [2,3,4]. Many commonly used orthopedic surgical materials, including nickel, cobalt, and chromium in metal alloys, as well as polymethyl methacrylate (PMMA) in bone cement, have been associated with these hypersensitivity reactions [1]. Accordingly, biocompatibility remains a crucial consideration in implant selection, implementation, and monitoring of surgical implants.

Among thousands of metallic materials, titanium-based implants are among the most common for orthopedic surgeries. Due to its mechanical strength, corrosion resistance, chemical stability, fatigue strength, low modulus, lightweight, reduced artifact generation in magnetic resonance imaging (MRI), and biocompatibility [5], titanium has been used in orthopedic surgeries, including total hip arthroplasty (THA), total knee arthroplasty (TKA), and fracture fixation [6,7,8,9]. A systematic review reported that titanium plates, compared to stainless steel implants, showed lower failure rates and fewer complications despite equal or lower biomechanical properties [10].

Nevertheless, the biocompatibility of titanium-based implants has been challenged in recent years, following a growing number of reports of bone loss, titanium implant failures, and titanium toxicity [11]. Although titanium is generally considered an inert material, allergic reactions to titanium-based implants may still occur, with various manifestations. Some reports have presented the possible allergic reaction after titanium osteosynthesis and found symptom improvement after implant removal, suggesting a possible causal relationship between the implant and the symptoms [12,13].

Currently, there is no single definitive test for titanium hypersensitivity, and diagnostic strategies remain heterogeneous and largely supportive rather than definitive [14]. Patch testing is the most commonly used examination for suspected metal hypersensitivity, but its clinical utility for titanium is controversial because test series and reagents are not standardized and may not adequately capture titanium-related sensitization [15,16,17]. Consequently, surgeons often combine the overall clinical course with available supportive tests rather than relying on only one test result to establish the diagnosis.

One important diagnostic challenge is that implant-related hypersensitivity can be similar to common postoperative complications like fracture-related infection (FRI) with overlapping manifestations like pain, local swelling, or delayed wound healing [18]. Because FRI is serious and more common in trauma orthopedic surgery, current consensus recommendations emphasize standardized definitions and structured diagnostic criteria to exclude other potential diagnoses [19].

This case report presents three cases of suspected hypersensitivity to titanium-based implants, emphasizing their clinical manifestations, different diagnostic processes, and management outcomes.

## 2. Case Presentation

The case report presents three patients with different sites of fractures (Table 1), involving two female and one male patients, who underwent removal of a titanium-based plate in a regional hospital in Taiwan. Before the removal of the plates, all patients had previously undergone Open Reduction and Internal Fixation (ORIF) of fractures using titanium-based plates, specifically “Ti-6Al-4V” (90% titanium, 6% aluminum, 4% vanadium, 0.25% iron, and 0.2% oxygen). The manifestations and the radiographs of the operative site are summarized in Table 2.

### 2.1. Case 1

**Figure 1:**
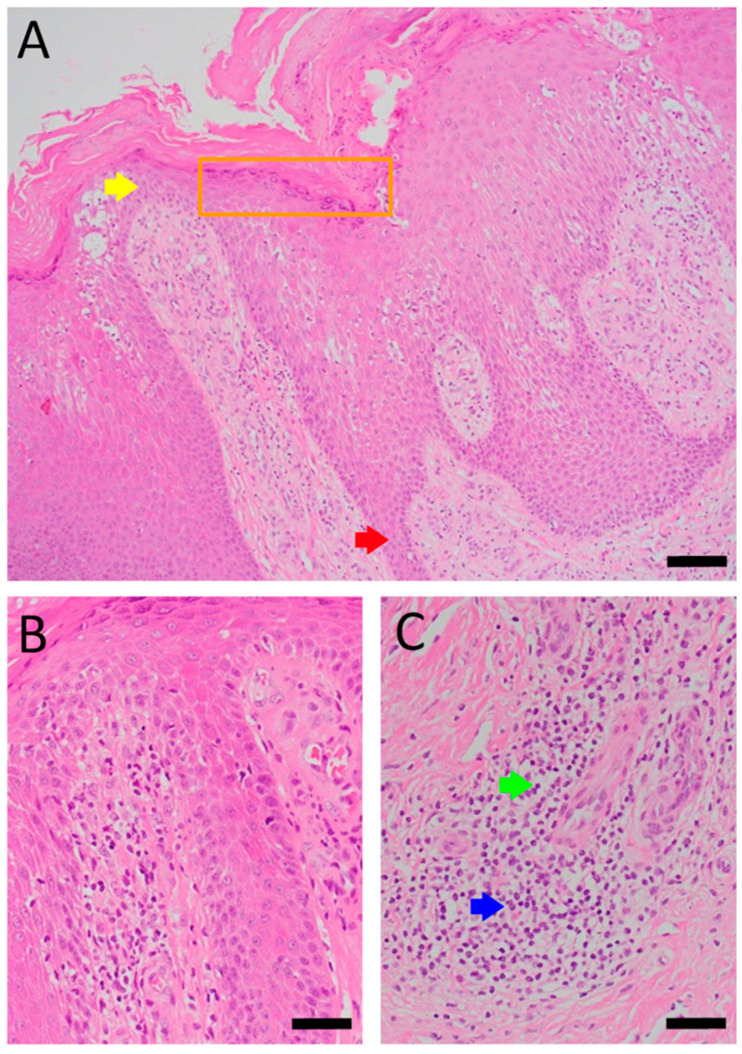
Histopathology of Patient A’s skin lesion showing psoriasiform dermatitis. Sections of Patient A’s skin lesion (**A**) demonstrate epidermis changes, including acanthosis, hyperkeratosis, parakeratosis, and spongiosis. Alternating zones of hypo- and hypergranulosis (orange box), elongation of rete ridges (red arrow), and thinning of suprapapillary plates (yellow arrow) are also noted. Higher magnification views (**B**,**C**) demonstrate dermal perivascular and intra-epidermal infiltration by neutrophils (blue arrow) and lymphocytes (green arrow). However, eosinophils are rarely seen (Scale bar: (**A**): 100 μm, (**B**,**C**): 50 μm).

Patient A was a 70-year-old female with a right distal tibiofibular fracture and underwent ORIF in March 2020. There were no known comorbidities for Patient A. One year after ORIF, she began to develop recurrent bullae formation of the right foot, accompanied by tenderness around her operative wound. Neuromuscular function and range of motion were intact, but a local skin rash and bullae formation with hyperpigmentation were observed. The white blood cell count was within the normal range at 3739/μL, which reduced the possibility of FRI. A biopsy of the skin lesion from Patient A was evaluated for pathological examination (Figure 1). The lesion showed psoriasiform dermatitis changes. The epidermis demonstrated acanthosis, hyperkeratosis, parakeratosis, and spongiosis. There were alternating zones of hypo- and hypergranulosis, elongation of rete ridges, and thinning of suprapapillary plates. Neutrophils and lymphocytes infiltrated the epidermis and around blood vessels in the dermis, but eosinophils were rarely seen, which was less typical of an allergic reaction. However, delayed-type hypersensitivity (type IV) was suggested given the chronic 2-year course, manifestations without pus or highly elevated white blood cell counts, and a biopsy-proven predominant presence of neutrophils and lymphocytes. A topical corticosteroid was prescribed to manage the symptoms, but only a slight improvement was observed. Based on her medical history, as well as physical and pathological examinations, a diagnosis of contact dermatitis due to suspected hypersensitivity to the titanium alloy in osteosynthesis was suggested. Radiographs showed bone unions, and fixation with the titanium alloy was no longer necessary. Therefore, she underwent plate and screw removal in March 2023. The symptoms of allergy were resolved after implant removal following confirmed bone unions.

### 2.2. Case 2

Patient B was a 60-year-old male with a right distal fibular fracture and underwent ORIF in October 2019. His medical history included diabetes mellitus, hypertension, chronic kidney disease stage 2, and benign prostatic hyperplasia under regular medical control. He developed continuous painless right lateral ankle swelling of the right ankle for 6 months after the ORIF. His physical examination showed a local skin rash with progressive hyperpigmentation and hyperkeratosis around the operative site and local lymph edema at the right ankle. No fever, pus formation, or other specific signs were noted, and the white blood cell count of 8690/μL was also within the normal range before the removal of the implant. Accordingly, FRI was considered less likely, and an impression of hypersensitivity to the titanium plate was suspected. Radiographs confirmed bone unions over the right distal fibula. Under the impression of a chronic allergic reaction to the plates, he underwent the removal of the implants 6 months after ORIF. During surgery, screw loosening was noted, which may be related to chronic inflammatory reactions. The allergic manifestations resolved after the removal of the implants.

### 2.3. Case 3

**Figure 2:**
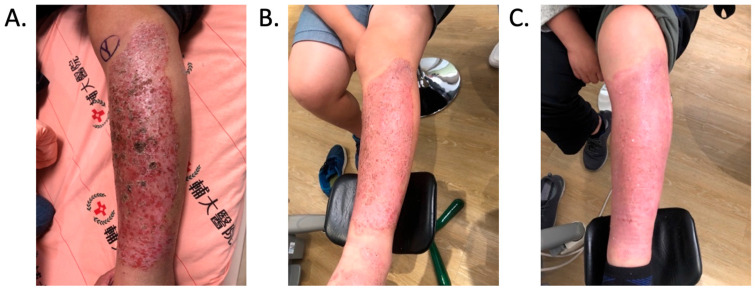
Improvement in the cutaneous allergic symptoms for Patient C after implant removal. (**A**) Clinical appearance before implant removal. Non-English text visible in the photograph is unrelated to the clinical findings. (**B**) Clinical appearance at the first follow-up in 2 months after implant removal. (**C**) Postoperative appearance at the 1-year follow-up after implant removal. The symptoms improved after titanium-removal surgery.

Patient C was a 42-year-old female with a left tibial fracture and underwent ORIF in February 2019. There were no known comorbidities for her. Four months after ORIF, she developed increasing tenderness, a local skin rash, a nonhealing wound, and intermittent clear discharge on the left leg. Physical examination revealed bullae formation, scaling with an erythematous base within a well-defined margin. Range of motion, distal motor function, circulation, and sensory function were intact. Radiographs confirmed a union of the tibia at the fracture site. There was no purulent drainage or fever, and the white blood cell count was 11,900/μL, which was not sufficient to confirm infection in the absence of purulence or systemic signs. She underwent a patch test for hypersensitivity, and the result showed an allergy to metal complexes. Under the impression of suspected hypersensitivity to the implants, she underwent the removal of the implants. The allergic symptoms were resolved after the implant removal. Follow-up at two months and one year showed improvement in symptoms (Figure 2).

## 3. Discussion

Hypersensitivity to implants is uncommon but important because its clinical presentations can resemble more common postoperative complications. Current reviews of hypersensitivity to orthopedic implants state that most allergic reactions are related to Type IV (delayed) hypersensitivity, and manifestations include localized dermatitis, pruritus, edema, pain, delayed wound or fracture recovery, persistent secretion, and implant failure [2,20,21]. Mechanistically, implant wear and corrosion may lead to the release of titanium-containing particles or metal ions, which can act as immunologic triggers [22,23]. These stimuli may promote a T-cell-mediated response, including activation of CD4+ Th1 lymphocytes and subsequent production of pro-inflammatory cytokines, contributing to delayed and persistent local inflammation [22,23]. Although cutaneous sensitivity to metal in the general population is common, at about 10–15%, deep-tissue allergic reactions due to implants are relatively rare [2,22]. Even in patients with confirmed metal allergy by patch tests, there were only about 17.6% of patients who were confirmed with metal hypersensitivity [24].

In this case report, three patients developed persistent cutaneous reactions around the operative site months after ORIF with Ti-6Al-4V implants, consisting of 90% titanium, 6% aluminum, 4% vanadium, 0.25% iron, and 0.2% oxygen. Titanium was the major constituent of the alloy, and it should be less likely to trigger an allergic reaction. In retrospective studies with 100 patients over 8 years, titanium-related allergy (1%) has been reported less common compared to nickel (23%), amalgam (15%), palladium (14%), copper (9%), cobalt (9%), mercury (4%), tin (2%), vanadium (2%), gold (2%), and molybdenum (1%) [25]. Nonetheless, rare titanium-related hypersensitivity was still possible and was recognized in various specialties with manifestations like eczema, bullous formation, localized pruritus, dermatitis, and swelling/edema [21]. These symptoms, along with additional patch test or other testing results, supported titanium as a possible cause, especially when the symptoms persist or progress despite typical postoperative wounds and anti-inflammatory management [21].

Current difficulties are that confirming titanium-alloy hypersensitivity still relies on diagnosis of exclusion, especially after ORIF, where FRI and other mechanical dysfunctions are more common. Current FRI consensus focuses on structured assessment combining clinical manifestations, laboratory tests, imaging, and microbiology or histology examinations [19,26]. Using the suggestive criteria in this consensus [19], three patients in this case report should be considered less likely for infections because they lacked purulence or abscess, systemic infectious symptoms, non-union bone, and evidence of highly elevated leukocytosis or infectious pathological reactions. In addition, radiographs reported bone unions, excluding any other non-union inflammation. However, some inflammation reactions may resemble FRI, so detailed documentation of wound appearance, symptom progression, laboratory results, or imaging is important before a confirmed diagnosis of allergy.

Current confirming tests for titanium-related allergy are also not entirely feasible. Patch testing is commonly used to evaluate possible type IV metal hypersensitivity, but standard patch tests often focus more on common allergens such as nickel or cobalt rather than titanium. Several common patch tests include the U.S. Food and Drug Administration-approved T.R.U.E. Test (Thin-Layer Rapid Use Epicutaneous) [27], the expanded American Contact Dermatitis Society’s Core Panel (2020 updated) [28], and the European Baseline Series [29]. None of these patch tests includes titanium as a common allergen [27,28,29], which leads to a difficult diagnosis of titanium-alloy hypersensitivity. Moreover, even though a titanium allergen test was included, the sensitivity was still low, supported by a current retrospective study showing insufficient sensitivity using titanium dioxide, one of the most common test agents [16]. Other assays like intradermal tests, lymphocyte transformation test (LTT), lymphocyte migration inhibition test, lymphocyte activation test, triple assay, and bidigital O-ring test (BiDORT) may assist in providing evidence, but their availability was limited by costs, preservation measures, or other factors [21,30]. Emerging laboratory tests like nanoparticulate and ionic titanium antigens would also have the potential for predicting hypersensitivity testing [31], but they are not yet included in the routine testing.

These limitations were reflected across our three cases because allergy testing was heterogeneous, and each modality has distinct constraints. For Patient A, histopathology provided supportive evidence of a chronic inflammatory dermatosis and helped exclude infection, but it is not specific for titanium-alloy hypersensitivity and cannot identify the causal metal. For Patient B, a clinical diagnosis of exclusion is pragmatic after infection and mechanical failure are ruled out through laboratory tests, but it remains indirect and may be vulnerable to residual diagnostic uncertainty. For Patient C, patch testing can support delayed-type metal sensitization, but the result was not titanium-specific. Accordingly, no single approach serves as a standalone gold standard, and these findings should be interpreted as complementary, supportive evidence within the overall clinical context.

In clinical settings, once bone union was confirmed without identifying other etiologies, implant removal could be both diagnostic and therapeutic, as demonstrated by improvements in allergic symptoms for all patients. In these three cases, hypersensitive symptoms resolved after the implant removal, which suggested a suspected titanium-alloy hypersensitivity reaction. Similar symptom resolution after plate removal from bone shortening surgery was presented in a case report with a suspected titanium allergy [32].

Collectively, this case report demonstrated a practical diagnostic and management approach for titanium-alloy hypersensitivity from excluding FRI-related infection and other etiologies, using patch test and pathology tests to provide evidence when available, and finally considering implant removal after bone unions to resolve the symptoms.

## 4. Conclusions

This case report presents three rare cases with delayed and persistent cutaneous reactions after implantation of titanium-alloy implants that resolved after implant removal. Given the absence of diagnostic criteria for titanium allergy, suspected titanium alloy hypersensitivity, like an extended eczematous or bullous dermatitis after ORIF, should be considered a diagnosis of exclusion with supportive tests when available. If symptoms persist after conservative management and bone union is confirmed, implant removal can be considered as a diagnostic–therapeutic option. It emphasizes the importance of clinical reasoning with a clear clinical course and symptoms and supports the need for improved diagnostic tools and standardized diagnostic criteria.

## Figures and Tables

**Table 1:** Demographic data of the three patients.

No.	Age	Sex	Primary Diagnosis	Comorbidities	OP	Original OP Date	Symptoms/Reactions	OP Removal Date	Implant Removal Operative Time
A	70	F	Right distal tibiofibular fracture	Denied	ORIF	March 2020	Tenderness, skin rash, and bullae formation	March 2023	2:10:00
B	60	M	Right fibular fracture	DM, HTN, CKDS2, BPH		October 2019	Painless, local lymphedema, skin rash, hyperkeratosis, and hyperpigmentation	March 2020	1:10:00
C	42	F	Left tibial fracture	Denied		February 2019	Tenderness, skin rash, bullae formation, nonhealing wound with intermittent clear discharge, and scaling with an erythematous base	December 2019	1:48:00

OP: Operation; F: Female; M: Male; DM, Diabetes Mellitus; HTN, Hypertension; CKDS2, Chronic Kidney Disease Stage 2; BPH: Benign Prostatic Hyperplasia. ORIF: Open Reduction and Internal Fixation.

**Table 2:** Clinical appearance and radiographic images before and after titanium-alloy implant removal.

Patient	A	B	C
Local Allergic Reactions			
Pre-Operation	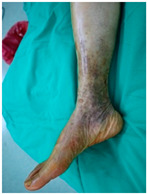	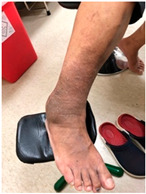	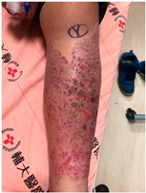
Radiographs			
Pre-Operation	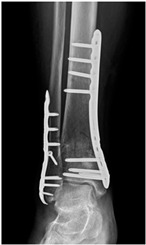	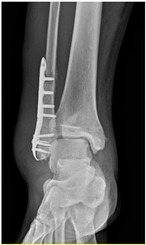	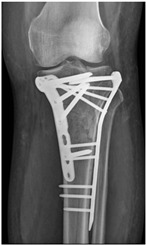
Post-Operation	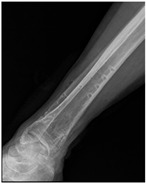	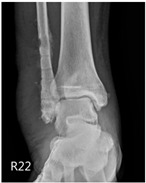	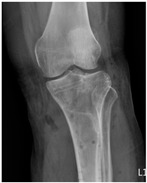

Patient C: Non-English text visible in the photograph is unrelated to the clinical findings.

## Data Availability

The authors declare that the data supporting the findings of this study are available within the paper.

## Abbreviations

The following abbreviations are used in this manuscript:

ORIF	Open Reduction and Internal Fixation
Ti-6Al-4V	Titanium–Aluminum–Vanadium alloy (Ti 90%, Al 6%, V 4%)
FRI	Fracture-Related Infection
PMMA	Polymethyl Methacrylate
MRI	Magnetic Resonance Imaging
THA	Total Hip Arthroplasty
TKA	Total Knee Arthroplasty
OP	Operation
F	Female
M	Male
DM	Diabetes Mellitus
HTN	Hypertension
CKDS2	Chronic Kidney Disease Stage 2
BPH	Benign Prostatic Hyperplasia
T.R.U.E. Test	Thin-Layer Rapid Use Epicutaneous Test
BiDORT	Bi-Digital O-Ring Test
